# Arecoline Enhances Phosphodiesterase 4A Activity to Promote Transforming Growth Factor-β-Induced Buccal Mucosal Fibroblast Activation via cAMP-Epac1 Signaling Pathway

**DOI:** 10.3389/fphar.2021.722040

**Published:** 2021-11-08

**Authors:** Bo Zhang, Lihua Gao, Chunsheng Shao, Mingsi Deng, Liangjian Chen

**Affiliations:** ^1^ Department of Stomatology, The Third Xiangya Hospital of Central South University, Changsha, China; ^2^ Department of Dermatology, The Third Xiangya Hospital of Central South University, Changsha, China; ^3^ Department of Orthodontics, Changsha Stomatological Hospital, Changsha, China

**Keywords:** oral submucous fibrosis, buccal mucosal fibroblast, arecoline, PDE4A, cAMP-Epac1 signaling pathway

## Abstract

Chewing areca nut (betel quid) is strongly associated with oral submucous fibrosis (OSF), a pre-cancerous lesion. Among the areca alkaloids, arecoline is the main agent responsible for fibroblast proliferation; however, the specific molecular mechanism of arecoline affecting the OSF remains unclear. The present study revealed that arecoline treatment significantly enhanced Transforming growth factor-β (TGF-β)-induced buccal mucosal fibroblast (BMF) activation and fibrotic changes. Arecoline interacts with phosphodiesterase 4A (PDE4A) to exert its effects through modulating PDE4A activity but not PDE4A expression. PDE4A silence reversed the effects of arecoline on TGF-β-induced BMFs activation and fibrotic changes. Moreover, the exchange protein directly activated by cAMP 1 (Epac1)-selective Cyclic adenosine 3′,5′-monophosphate (cAMP) analog (8-Me-cAMP) but not the protein kinase A (PKA)-selective cAMP analog (N6-cAMP) remarkably suppressed α-smooth muscle actin(α-SMA) and Collagen Type I Alpha 1 Chain (Col1A1) protein levels in response to TGF-β1 and arecoline co-treatment, indicating that cAMP-Epac1 but not cAMP-PKA signaling is involved in arecoline functions on TGF-β1-induced BMFs activation. In conclusion, arecoline promotes TGF-β1-induced BMFs activation through enhancing PDE4A activity and the cAMP-Epac1 signaling pathway during OSF. This novel mechanism might provide more powerful strategies for OSF treatment, requiring further *in vivo* and clinical investigation.

## Introduction

Oral submucous fibrosis (OSF) is a pre-cancerous lesion related to chewing areca nut (betel quid). This custom is common among people in South Asia and has now spread to Europe and North America. OSF incidence is relatively high; besides, it may also cause cancer-related death once it develops into squamous cell carcinoma (SCC). The combination of betel quid and tobacco significantly increased OSF incidence (Haider et al., 2000; More and Rao, 2019; Rao et al., 2020). In contrast, smoking and chewing betel nut have been demonstrated to be independent risk factors for cancers of the esophagus, mouth, and throat (Ko et al., 1995; Znaor et al., 2003).

The main components of areca oil, arecoline from areca nuts and copper, lead to fibroblasts’ dysfunction and fibrotic changes (Meghji et al., 1997; Wollina et al., 2015). Among the areca alkaloids such as arecoline, arecadine, guvacoline, and guvacine, arecoline is the main agent responsible for fibroblast proliferation. Under the influence of slaked lime (Ca(OH) 2), arecoline gets hydrolyzed to arecadine, which displays a remarkable role in fibroblasts (Tilakaratne et al., 2006). Depleting cellular glutathione (GSH) by arecoline predisposes the oral mucosal fibroblasts to various genotoxic and cytotoxic stimulation (Shieh et al., 2003). Chang et al. (Chang et al., 2014) revealed that several molecules, including plasminogen activator inhibitor-1 (Yang et al., 2003), insulin-like growth factor-1 (IGF-1) (Tsai et al., 2005), and nuclear factor kappa-light-chain-enhancer of activated B cells (NF-κB) (Ni et al., 2007), could be expressed in human buccal mucosal fibroblasts (BMFs) after treatment with arecoline. Moreover, arecoline led to vimentin expression (Chang et al., 2002) in human BMFs. Notably, the upregulation of these factors mentioned above might contribute to the extracellular matrix (ECM) accumulation in OSF (Chang et al., 2002; Yang et al., 2003; Ni et al., 2007; More et al., 2020). However, the specific molecular mechanism by which arecoline affects the OSF remains unclear.

In the present study, primary normal (BMFs were isolated from oral submucous fibrosis tissues and identified; transforming growth factor-β1 (TGF-β1) stimulation was conducted to activate the BMFs, as confirmed by TGF-β1 signaling activation and increases in α-smooth muscle actin(α-SMA) and Collagen Type I Alpha 1 Chain (Col1A1) protein levels. Firstly, we examined how arecoline affected TGF-β1-caused BMFs activation. Secondly, as for the molecular mechanism, the study performed Protein-Protein Interaction analyses (STRING analysis) to identify candidate proteins that might interact with arecoline. Thirdly, we performed Kyoto encyclopedia of genes and genomes (KEGG) signaling pathway annotation and gene ontology (GO) target genes function significance analyses to identify the signaling pathways in which the candidate proteins might be enriched. These bioinformatics analyses showed that PDE4A might interact with arecoline, and proteins that interact with arecoline were enriched in the cAMP signaling. After that, the dynamic effects of arecoline and candidate factors on TGF-β1-induced BMFs activation and the involved signaling pathways were investigated. In summary, the study provided a novel mechanism by which arecoline enhances TGF-β1-induced BMFs activation, promoting OSF progression.

## Materials and Methods

### Clinical Sample Collection and Histological Analysis

Six normal buccal mucosa smaples were obtained from healthy volunteers (age is 46 ± 14.4 years, F/M is 1/5) who did not have areca nut chewing habits. Six OSF buccal mucosa samples were obtained from OSF patients (the clinical stage is II-IV, age is 47.5 ± 15.1 years, F/M is 0/6). The experimental protocols were approved by the ethics committee of the Third Xiangya Hospital, Central South University, Ethics No. 2019-S061.

The clinical samples were fixed with 4% paraformaldehyde, embedded in paraffin, and sliced into sections of 4-μm thick. Perform H&E staining to evaluate the histopathological features (Qin et al., 2019). The expression of PED4A was determined by Immunohistochemical (IHC) staining as previously described method (Wang et al., 2020).

### Buccal Mucosa Fibroblasts Isolation and Identification

Primary normal BMFs from oral submucous fibrosis tissues were isolated and cultivated following the methods described before (Chang et al., 1998; Chang et al., 2014). BMFs were identified by immunofluorescent staining (IF) using the Vimentin antibody (ab92547, Abcam, Cambridge, MA, United States). BMFs were cultured in Dulbecco’s Modified Eagle Medium (DMEM) (GBICO, Waltham, MA, United States) containing 10% fetal bovine serum (FBS; GBICO) and treated with 5 ng/ml TGFβ1 (R&D Systems, Inc., Minneapolis, MN, United States) for 24/48 h or co-treated with 10, 20 and 50 μg/ml arecoline (Aladdin, China) for 48 h. Then, cells were harvested for further experiments. For PDE4A inhibition, a selective inhibitor of PDE4 rolipram was used (Ding et al., 2018). Cells were treated with 25 nM rolipram for 48 h.

### Bioinformatics Analysis

The BATMAN-TCM (Liu et al., 2016) (http://bionet.ncpsb.org.cn/batman-tcm/) was used to predict the arecoline interacted proteins (top 200 proteins with highest interaction score, *p* < 0.05). Next, the KEGG signaling pathway annotation and GO target genes function significance analyses were performed on candidate proteins that might interact with arecoline using STRING-DB (Protein-Protein Interaction Networks Functional Enrichment Analysis) (https://string-db.org/) (Szklarczyk et al., 2015). Gene Expression Omnibus (GEO) dataset GSE107591 (expression profiling of 23 normal and 24 head and neck squamous cell carcinoma (HNSCC) tissues) was downloaded using R language GEOquery package (Davis and Meltzer, 2007), and the differential expression genes were analyzed by Limma package (Ritchie et al., 2015). PDE4A expression was analyzed based on TCGA-HNSCC data using the online tool UALCAN (http://ualcan.path.uab.edu/index.html) (Chandrashekar et al., 2017).

### Immunoblotting

The protein levels of α-SMA, COL1A1, Smad2, p-Smad2, PED7A, PED4A, PKA, p-PKA, total Ras-proximate-1 (Rap1), Guanosine-5′-triphosphate (GTP)-Rap1, and exchange protein directly activated by cAMP 1 (Epac1), cAMP-response element-binding protein (CREB) and p-CREB were examined by immunoblotting following the methods described before (Liu et al., 2018) using the antibodies listed below: anti-α-SMA (ab5694, Abcam), anti-COL1A1 (ab34710, Abcam), anti-Smad2 (ab40855, Abcam), anti-p-Smad2 (ab53100, Abcam), anti-PDE4A (ab14607, Abcam), anti-PDE4B (ab170939, Abcam), anti-PDE4C (ab170939, Abcam), anti-PDE4D (ab171750, Abcam), anti-PKA (BS-0520R, Woburn, MA, United States), p-PKA (ab75991, Abcam), anti-GTP-Rap1 (ab32373, Abcam), anti-Rap1 (ab14404, Abcam), anti-Epac1 (ab109415, Abcam), anti-CREB (ab31387, Abcam), anti-p-CREB (ab32096, Abcam), and anti-Glyceraldehyde 3-phosphate dehydrogenase (GAPDH) (ab8245, Abcam) and then with HRP-conjugated secondary antibody. Enhanced chemilumescent (ECL) substrates (Millipore, MA, United States) were used for signals visualization using GAPDH as an endogenous protein for normalization.

### Collagen Contraction Assay

BMFs were co-treated with 5 ng/ml TGFβ1 and 10, 20, or 50 μg/ml arecoline for 48 h, and the gel contraction ability of fibroblasts was determined by the collagen contraction assay following the methods described before (Yu et al., 2016) using collagen solution obtained from Sigma-Aldrich (2 mg/ml) (St. Louis, MO, United States). Contraction of the gels was photographed, and the areas were measured using ImageJ software (NIH, Bethesda, MD, United States) (Yu et al., 2013a).

### *In vitro* Migration Assays

BMFs were treated as described above and examined for migration capacity. The migration capacity of BMFs was examined using Transwell assay following the methods described before (Luo et al., 2019). The nonmigratory BMFs in the top chambers were removed with cotton swabs. The migrated BMFs on the lower membrane surface were fixed and stained with Crystal violet for nuclear staining (Beyotime, Shanghai, China). BMFs were counted under a microscope.

### Wound Healing Assay

The wound-healing assay was performed following the methods described before (Yu et al., 2016) using a 12-well culture dish and a sterile 200 μl plastic pipette tip to create a denuded area. Cell movement into the wound area was photographed at 0 and 48 h under a microscope (Yu et al., 2013b).

### cAMP Concentration Assay

BMFs were starved with serum-free DMEM media for 16–18 h, and then treated with eugenol for 7 min and lysed with 0.1 M HCl. cAMP levels were measured using the Direct cAMP ELISA kit (ADI-900-066, Enzo Life Sciences, Hong Kong, China).

### Extracellular PDE4A Activity Assay

PDE4A enzymatic activity was determined using a PDE4A assay kit (Cat. 60,340, BPS Bioscience Inc., United States). Procedures were performed according to manufacturer’s instructions. Briefly, 25 µl of FAM-cAMP (200 nM), 20 µl PDE buffer contained 80 pg PDE4A protein, and 5 µl arecoline were added to the microplate well and incubated for 1 h. Then, 100 µl binding agent was added to each well and incubated for 30 min with slow shaking. Finally, the each well was determined by a multifunction microreader (Bio-rad, United States) with excitation wavelength 485 nm and emission wavelength 528 nm.

### Statistical Analysis

Data are presented as mean ± SD. A Student’s *t-*test was used to compare the continuous variables between two groups. The analysis of variance (ANOVA) test followed Tukey post-hoc test was used to analyze the difference among more than two groups. Mann-Whitney U test was used for TCGA and GSE data analysis. *p* < 0.05 was considered statistically significant.

## Results

### Arecoline Enhances TGF-β1-Induced Activation of Buccal Mucosal Fibroblasts

**FIGURE 1:**
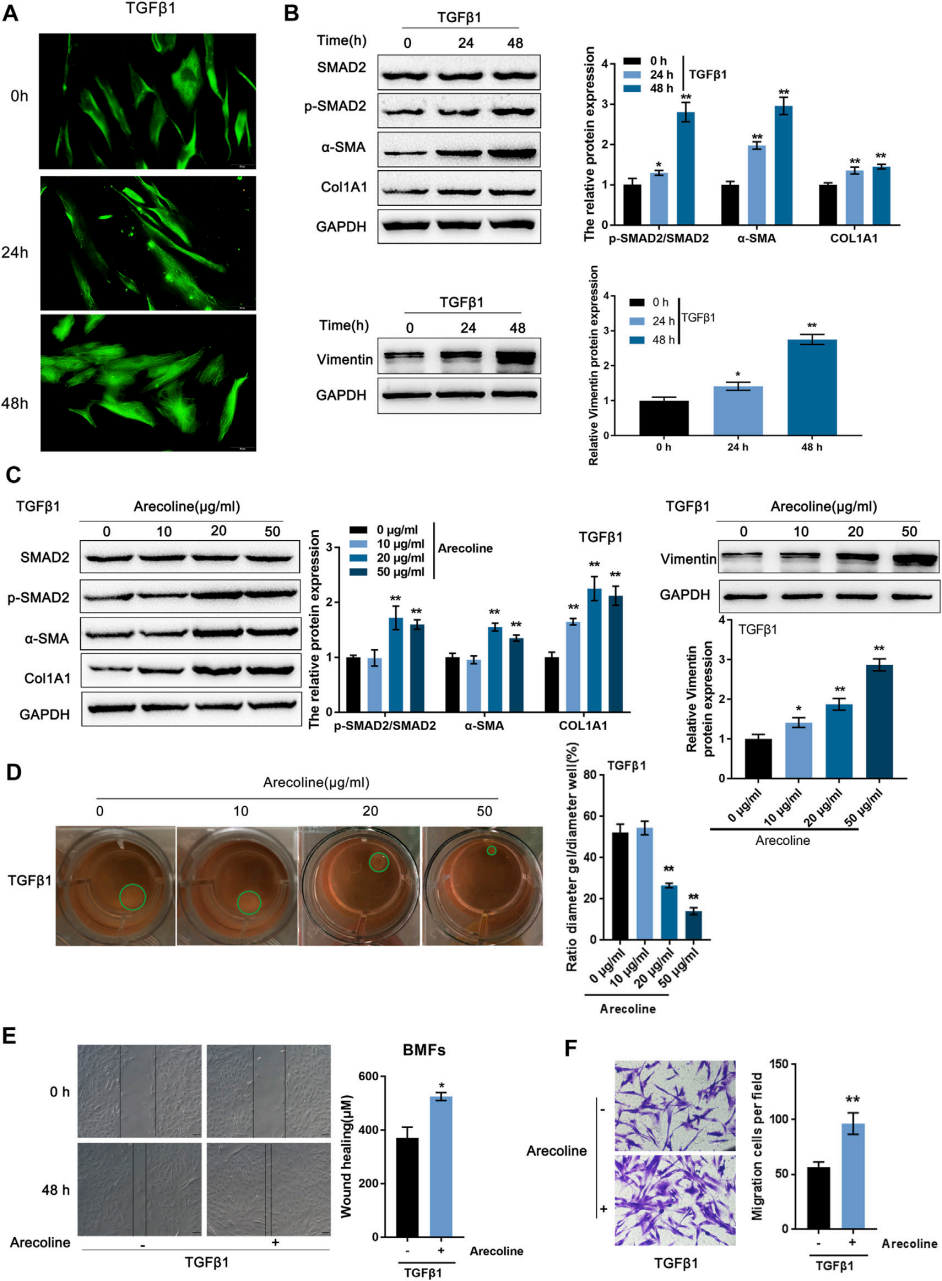
Arecoline enhances TGF-β1-induced activation of buccal mucosal fibroblasts (BMFs). **(A,B)** BMFs were isolated, treated with 5 ng/ml TGF-β1 for 0, 24, and 48 h, and identified for activation by **(A)** IF staining with Vimentin antibody and **(B)** Immunoblotting with Smad2, p-Smad2, α-SMA, Col1A1, and Vimentin. **(C,D)** Then, BMFs were co-treated with arecoline (0, 10, 20 or 50 µg/ml) and 5 ng/ml TGF-β1 for 48 h and examined for **(C)** the protein levels of Smad2, p-Smad2, α-SMA, Col1A1, and Vimentin; **(D)** the gel contraction capacity. *n* = 3, *p < 0.05, **p < 0.01 compared with control group. ANOVA followed by a Tukey post-hoc test. **(E,F)** BMFs were treated with 5 ng/ml TGF-β1 in the presence or absence of 20 µg/ml arecoline for 48 h and examined for the migration capacity by Wound healing and Transwell assays. *n* = 3, *p < 0.05, **p < 0.01 compared to TGF-β1 treated group. student *t*-test.

BMFs were isolated from oral submucous fibrosis tissues and then treated with 5 ng/ml TGF-β1 for 0, 24, and 48 h for BMFs activation, which was identified by increased Vimentin content as revealed by IF staining (Figure 1A). After 24 h of TGF-β1 stimulation, the enhanced Smad2 phosphorylation indicated that the activation of the TGF-β1 signaling pathway; meanwhile, TGF-β1 stimulation-induced upregulation of α-SMA, Col1A1, and vimentin protein levels, further indicating TGF-β1-caused BMFs activation (Figure 1B).

To examine how arecoline affected TGF-β1-caused BMFs activation, we co-treated BMFs with 5 ng/ml TGF-β1 and 0, 10, 20, or 50 µg/ml arecoline and examined Smad2, p-Smad2, α-SMA, Col1A1, and vimentin protein levels. p-Smad2, α-SMA, Col1A1, and vimentin protein levels were -increased by TGF-β1 stimulation, and further enhanced by 20 and 50 µg/ml arecoline (Figure 1C). Simultaneously, the gel contraction capacity of BMFs was significantly inhibited by 20 and 50 µg/ml arecoline upon TGF-β1 stimulation (Figure 1D). Furthermore, 50 µg/ml arecoline significantly enhanced the migration capacity of BMFs induced by TGF-β1 stimulation (Figures 1E,F). These findings indicate that the activation of BMFs could be promoted by TGF-β1 while further enhanced arecoline treatment.

### Identification of Proteins Interacting With Arecoline by Bioinformatics and Experimental Analyses

**FIGURE 2:**
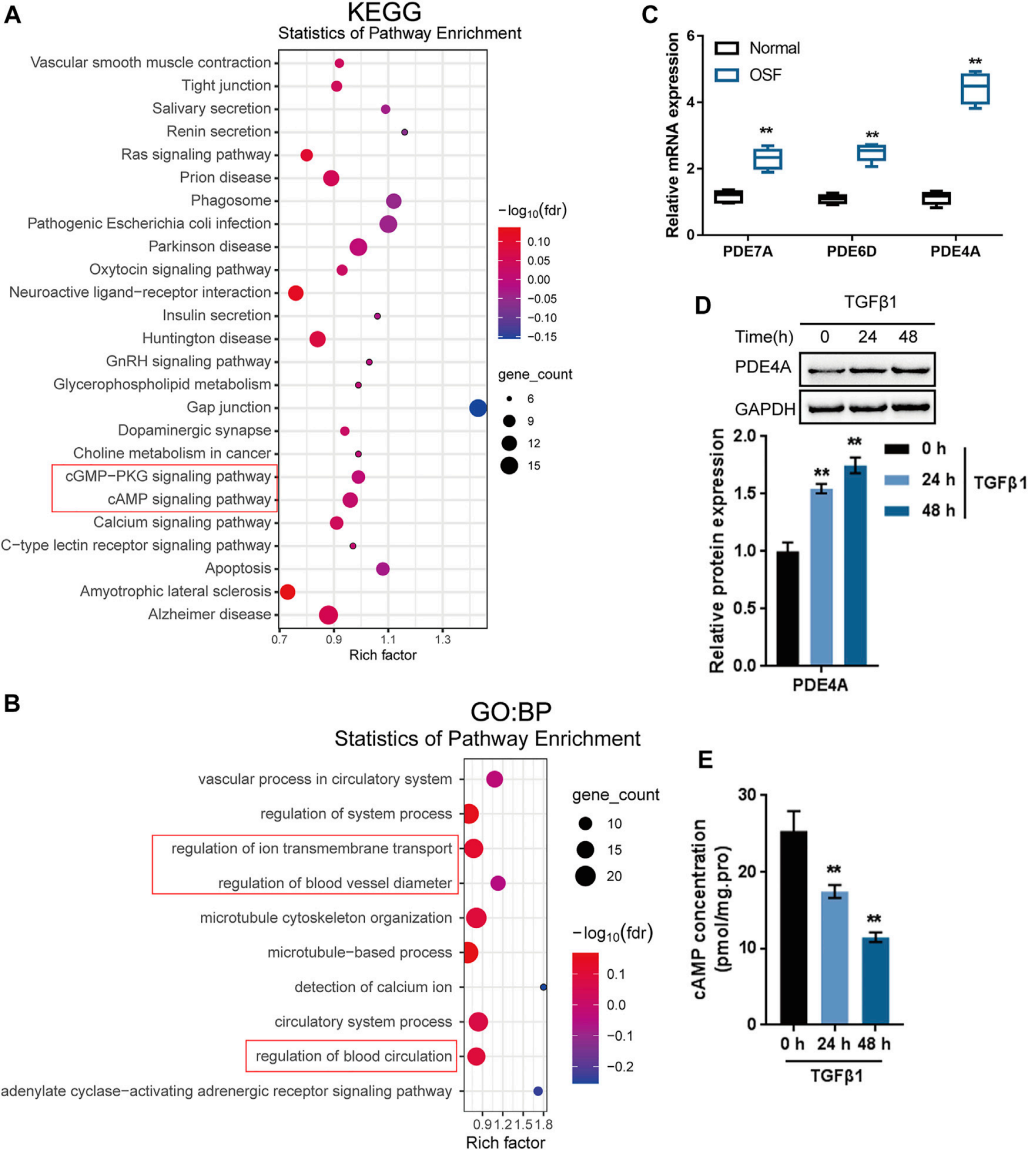
Identification of proteins interacting with arecoline by bioinformatics and experimental analyses. **(A,B)** KEGG signaling pathway annotation and GO target genes function significance analyses were performed on candidate proteins that might interact with arecoline. **(C)** The mRNA levels of PDE4A, PDE6D and PDE7A in collected normal oral mucosal and OSF tissues. **(D)** BMFs were treated with 5 ng/ml TGF-β1 for 0, 24, and 48 h and examined for the protein levels of PDE4A by Immunoblotting, *n* = 3. **(E)** cAMP concentration was determined by a direct cAMP ELISA kit. *n* = 3, **p < 0.01 compared with the normal or control group. ANOVA followed by Tukey post-hoc test.

Regarding the molecular mechanism of arecoline enhancing TGF-β1-induced BMFs activation, the study performed bioinformatics analyses. Through the Batman bioinformatics analysis, the top 200 proteins with the highest interaction score with arecoline (*p* < 0.05) were selected for KEGG signaling pathway annotation and GO target genes function significance analyses by online tool STRING. KEGG annotation analysis showed that these proteins were enriched in the cAMP and cGMP-PKG signaling pathways (Figure 2A). GO function analyses of biological process (BP) found that several biological processes were the related to cAMP pathway, including regulation of ion transmembrane transport (Csanády et al., 2019; Mihályi et al., 2020) regulation of blood vessel diameter (Waldkirch et al., 2010; Syed et al., 2019), and regulation of blood circulation (Gao and Raj, 2010) (Figure 2B). During the fibrosis progress, PDE/cAMP pathway could regulate renal fibrosis [(Ding et al., 2018)], lung fibrosis (Wójcik-Pszczoła et al., 2020), liver fibrosis (Essam et al., 2019) Moreover, BATMAN-TCM bioinformatics analysis showed that arecoline might directly interact with 22 PDE family members (Supplementary Table S1). These results indicate that arecoline might interact with the PDE proteins to exert its effects via the cAMP signaling pathway.

Next, OSF showed potential for malignant transformation (Ekanayaka and Tilakaratne, 2016). We further analyzed the PDE family expression in and TCGA-HNSCC and GSE datasets. The results showed that PDE4A, PDE6D and PDE7A were highly expressed in HNSCC compared to normal tissues (Supplementary Figure S1A). Moreover, among the three PDE family members, the fold change of PDE4A was the highest between the collected OSF tissues and normal control tissue (Figure 2C). A comparison of 23 clinical oral and throat cancers and 24 normal oral tissue samples (GSE107591) also reported that PDE4A was significantly up-regulated in tumor samples (Supplementary Table S2). IHC staining results showed that PDE4A levels in the collected OSF tissues were higher than normal buccal mucosa tissues (Supplementary Figure S1B). Therefore, PDE4A might be an important target gene involved in oral submucosal fibrosis and oral cancer.

To further confirm whether PDE4A is involved in the activation of BMFs, BMFs were treated with 5 ng/ml TGF-β1 for 0, 24, and 48 h and examined PDE4A protein levels and the cAMP concentration. The stimulation of TGF-β1 remarkably induced PDE4A protein expression (Figure 2D), whereas reduced the concentration of cAMP (Figure 2E) time-dependently. Thus, PDE4A is selected for further experiments.

### Arecoline Enhances TGF-β1-Caused BMFs Activation

**FIGURE 3:**
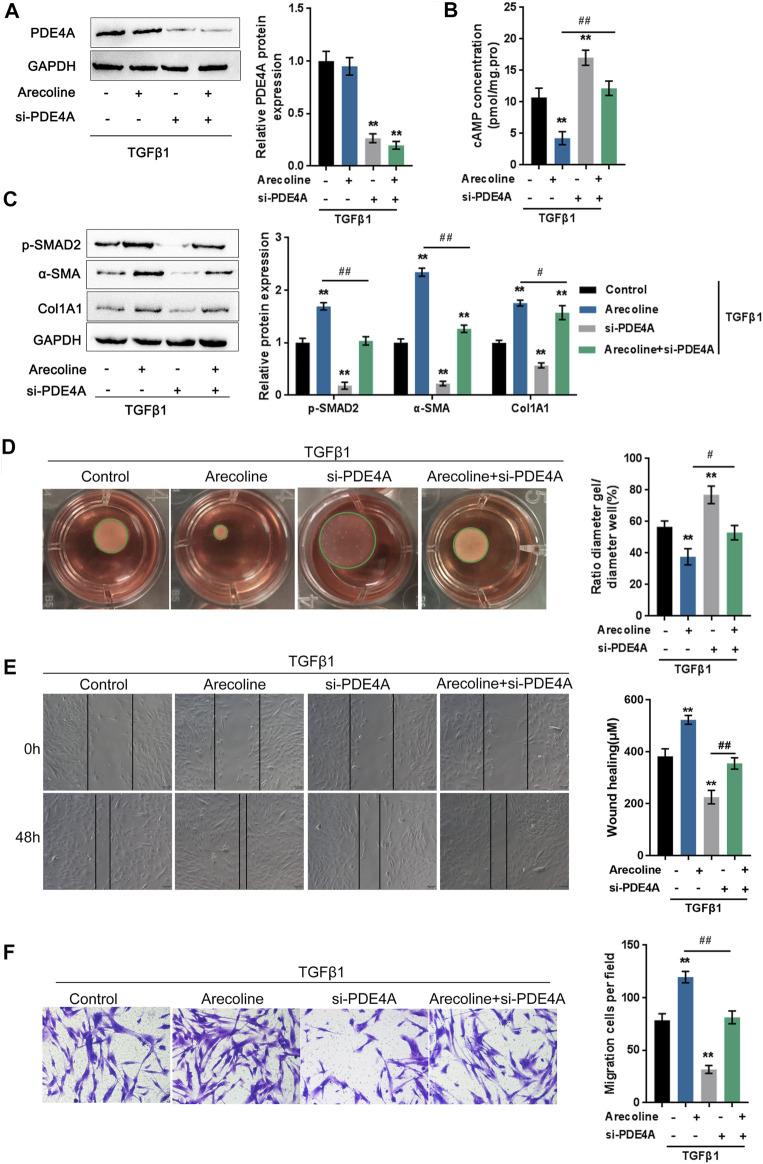
Arecoline enhances TGF-β1-induced BMFs activation. BMFs were transfected with si-PDE4A in the presence or absence of 50 µg/ml arecoline treatment upon 5 ng/ml TGF-β1 stimulation for 48 h, and then examined for **(A)** the protein levels of PDE4A by Immunoblotting, *n* = 3. **(B)** the cAMP concentration by ELISA, *n* = 3. **(C)** the protein levels of p-Smad2, α-SMA, and Col1A1 by Immunoblotting, *n* = 3. **(D)** the gel contraction capacity, *n* = 3. **(E,F)** the migration capacity by Wound healing and Transwell assays, *n* = 3. **p < 0.01, compared to control group; #*p* < 0.05, ##*p* < 0.01, compared to arecoline group. ANOVA followed by Tukey post-hoc test.

To validate the effects of PDE4A on arecoline function during TGF-β1-caused activation of BMFs, firstly, we determined the expression of PDE4A under arecoline treatment. Interestingly, arecoline did not affect PDE4A expression in the presence or absence of TGF-β1 (Supplementary Figure S2A and Figure 3A). But arecoline could directly increase the extracellular PDE4A activity (Supplementary Figure S2B). Under TGF-β1 stimulation, arecoline decreased cAMP concentration (Figure 3B), suggesting that arecoline may only affect the activity of PDE4A rather than its expression. Next, BMFs were transfected with si-PDE4A under TGF-β1 stimulation in the presence or absence of arecoline treatment and then evaluated the protein levels of PDE4A and the cAMP concentration as an indicator of PDE4A activity. As revealed by Immunoblotting, si-PDE4A transfection remarkably downregulated PDE4A protein levels upon TGF-β1 stimulation (Figure 3A). In the meantime, arecoline treatment significantly reduced, while PDE4A silence increased the cAMP concentration. The suppressive effect of arecoline on the cAMP concentration could be significantly reversed by PDE4A silence (Figure 3B).

Next, the study examined the dynamic effects of arecoline and PDE4A silence on TGF-β1-induced BMFs activation. As shown in Figure 3C, p-Smad2, α-SMA, and Col1A1 protein levels were up-regulated by TGF-β1 and further enhanced by arecoline treatment, while significantly decreased by PDE4A silence; the effects of arecoline treatment could be significantly reversed by PDE4A silence. Consistently, TGF-β1-caused suppression on the gel contraction capacity was further inhibited by arecoline treatment while rescued by PDE4A silence; the arecoline effect was also reversed by PDE4A silence (Figure 3D). Similarly, TGF-β1-induced migration capacity was further enhanced by arecoline treatment while suppressed by PDE4A silence; the effects of arecoline were reversed by PDE4A silence (Figures 3E,F). These data indicate that arecoline modulates the activity of PDE4A, but not its expression, during TGF-β1-induced BMFs activation.

### Arecoline Regulates TGF-β1-Induced BMFs Activation by Affecting the Activity of PDE4A

**FIGURE 4:**
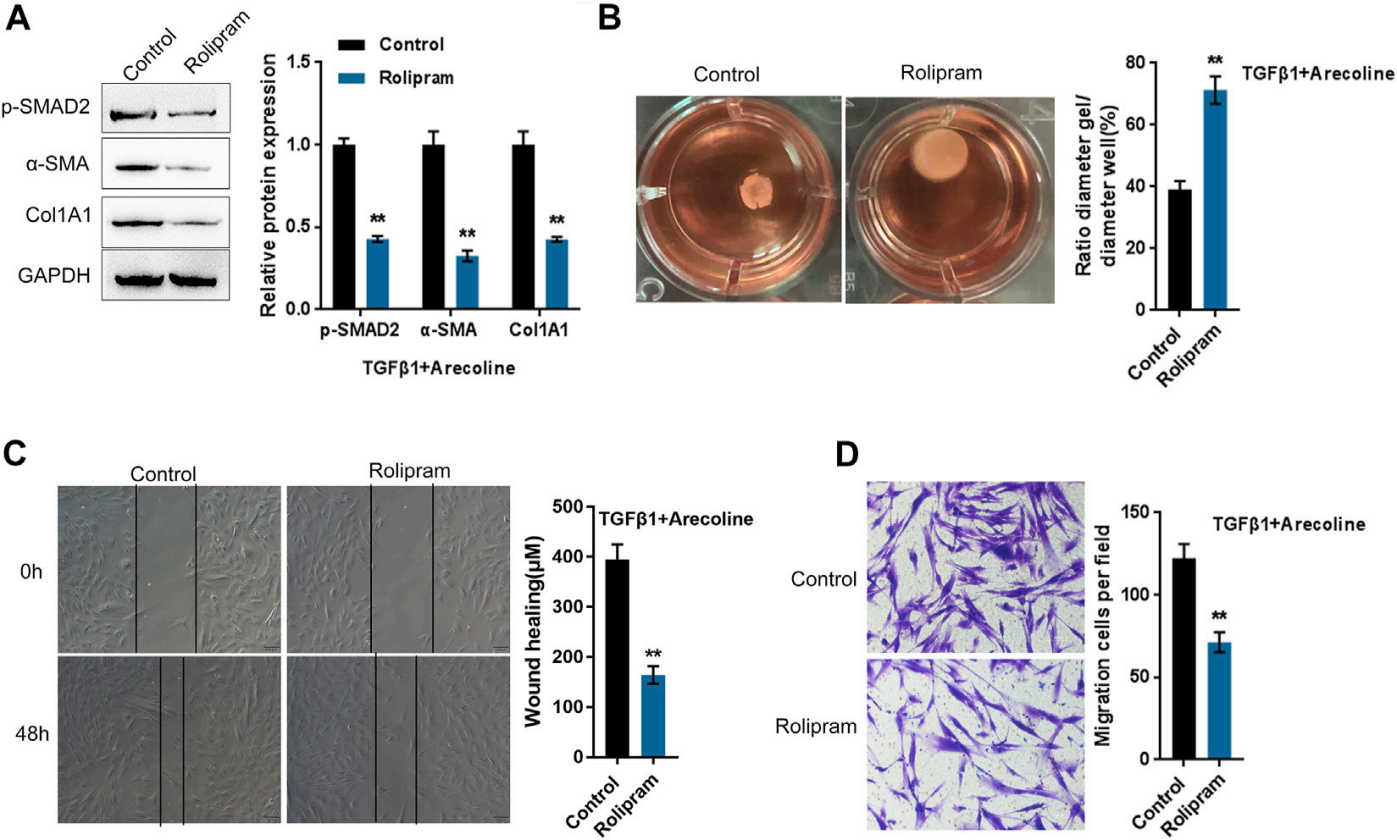
Arecoline regulates TGF-β1-induced BMFs activation by affecting the activity of PDE4A. BMFs were co-treated with 50 µg/ml arecoline and 5 ng/ml TGF-β1 in the presence or absence of PDE4 specific inhibitor Rolipram, and then examined for **(A)** the protein levels of *p*-Smad2, α-SMA, and Col1A1, *n* = 3; **(B)** the gel contraction capacity, *n* = 3. **(C,D)** the migration capacity by Wound healing and Transwell assays, *n* = 3 **p < 0.01 compared with the control group, student *t* test.

To further investigate whether arecoline affects PDE4A activity to modulate TGF-β1-induced BMFs activation, we co-treated BMFs with arecoline and TGF-β1 with or without PDE4 specific inhibitor rolipram, then evaluated for *p*-Smad2, α-SMA, and Col1A1 protein levels. According to Figure 4A, rolipram therapy remarkably inhibited p-Smad2, α-SMA, and Col1A1 protein levels. Consistently, rolipram treatment significantly increased the gel contraction capacity (Figure 4B) while inhibiting the migration capacity of the BMFs (Figures 4C,D). These data indicate that inhibiting PDE4A activity could attenuate the effects of arecoline on TGF-β1-induced BMFs activation.

### Arecoline Affects the Effects of PDE4A on the cAMP Pathway Upon TGF-β1 Stimulation

**FIGURE 5:**
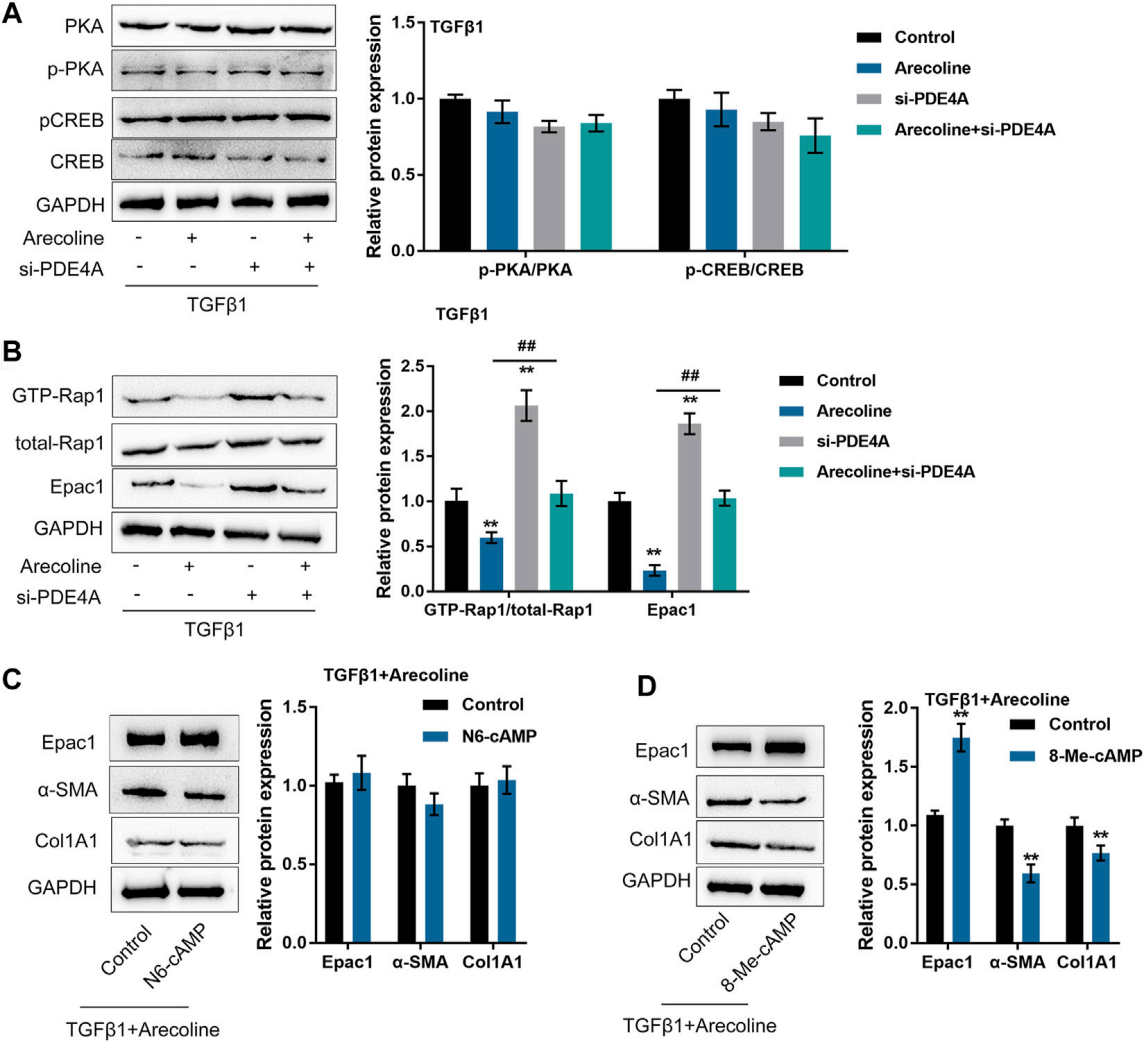
Arecoline affects the effects of PDE4A on the cAMP pathway upon TGF-β1 stimulation. BMFs were transfected with si-PDE4A in the presence or absence of 50 µg/ml arecoline treatment upon 5 ng/ml TGF-β1 stimulation, and then examined for **(A)** the protein levels of PKA, p-PKA, CREB, and p-CREB by Immunoblotting, *n* = 3. **(B)** the protein levels of GTP-Rap1, total Rap1, and Epac1 by Immunoblotting, *n* = 3. **p < 0.01, compared to the control group; ##*p* < 0.01, compared to arecoline group. ANOVA followed by Tukey post-hoc test. **(C)** BMFs were co-treated with arecoline and TGF-β1 in the presence or absence of PKA-selective cAMP analog N6-cAMP, and then examined for the protein levels of Epac1, α-SMA and Col1A1 by Immunoblotting, *n* = 3. **(D)** BMFs were co-treated with arecoline and TGF-β1 in the presence or absence of Epac1-selective cAMP analog 8-Me-cAMP, and then examined for the protein levels of Epac1, α-SMA and Col1A1 by Immunoblotting, *n* = 3. **p < 0.01, compared with the control group, student *t*-test.

PDE4 specifically catalyzes the hydrolysis of cAMP (Zhang, 2009); next, the study investigated whether arecoline could affect the effects of PDE4A on the cAMP signaling pathway in the process of TGF-β1-caused BMFs activation. We transfected BMFs with si-PDE4A with or without arecoline treatment upon the stimulation of TGF-β1, then evaluated the protein levels of the cAMP-PKA signaling factors (PKA, p-PKA, CREB, and p-CREB) and cAMP-Epac1 signaling factors (GTP-Rap1, total Rap1, and Epac1). As inferred from Figure 5A, the above-described treatment and transfection only caused moderate changes in the cAMP-PKA signaling factors. In contrast, arecoline treatment significantly decreased, while PDE4A silence up-regulated GTP-Rap1 and Epac1 protein levels; arecoline treatment effects could be significantly reversed by PDE4A silence (Figure 5B). Moreover, arecoline stimulation alone did not affect the expression of PDE4A and p-PKA/PKA ratio while decreased the Epac1 expression (Supplementary Figure S2), indicating that arecoline could inhibit the cAMP/Epac1 pathway. Altogether, these data suggest that the cAMP-Epac1 signaling but not the cAMP-PKA signaling might be involved in arecoline and PDE4A functions during TGF-β1-caused BMFs activation.

To further validate the above-described findings, we co-treated BMFs with arecoline and TGF-β1 with or without the PKA-selective cAMP analog (N6-cAMP) and then examined Epac1, α-SMA and Col1A1 protein levels. According to Figure 5C, under TGF-β1 stimulation, the application of N6-cAMP caused no significant changes in Epac1, α-SMA and Col1A1. Next, we co-treated BMFs with arecoline and TGF-β1 with or without the Epac1-selective cAMP analog (8-Me-cAMP), then evaluated α-SMA Col1A1 protein levels. Unlike N6-cAMP, 8-Me-cAMP significantly increased Epac1 protein levels and reduced α-SMA and Col1A1 protein levels (Figure 5D), indicating that cAMP-Epac1 signaling activation indeed suppressed TGF-β1-induced BMFs activation.

## Discussion

In the present study, arecoline treatment significantly promoted TGF-β-induced BMFs activation and fibrotic changes. Regarding the molecular mechanism, arecoline interacts with PDE4A to exert its effects through modulating PDE4A activity but not PDE4A expression. The effects of arecoline on TGF-β-induced BMFs activation and fibrotic changes could be reversed by PDE4A silence. Moreover, Epac1-selective cAMP analog 8-Me-cAMP but not PAK-selective cAMP analog N6-cAMP remarkably suppressed α-SMA and Col1A1 protein levels upon TGF-β1 and arecoline co-treatment, indicating that the cAMP-Epac1 but not cAMP-PAK signaling is involved in arecoline functions in TGF-β1-induced BMFs activation.

TGF-β1, as a multipotent cytokine, exerts pathological effects on organ fibrosis. After activating TGF-β1 in oral keratinocytes treated with arecoline integrin-αvβ6–dependently, human oral fibroblasts could transform into myofibroblasts (Moutasim et al., 2011). TGF-β-caused ZEB1 within smooth muscle cells led to α-SMA transcription via interacting with Smad3 and SRF (Nishimura et al., 2006). Chang et al. (2014) conducted the transdifferentiation of fibroblasts into myofibroblasts via treating BMFs with arecoline and provided a semblable mechanismα-SMA was up-regulated in a ZEB1-dependent manner. Moreover, betel quid extracts and arecoline have been reported to activate TGF-β signaling within human keratinocyte HaCaT cells (Khan et al., 2012) or activate TGF-β signaling, as well as α-SMA and COL1A1 expression within human gingival fibroblasts (Khan et al., 2012). Herein, the study observed similar results that 20 and 50 µg/ml arecoline treatment significantly enhanced TGF-β1 signaling activation and TGF-β1-induced BMFs activation, as manifested by increased p-Smad2, α-SMA, and Col1A1 protein levels, reduced gel contraction capacity, and enhanced migration capacity of BMFs.

Regarding the underlying mechanism, bioinformatics analyses indicate that PDE4A and cAMP signaling pathways might be involved in arecoline function in TGF-β1-induced BMFs activation and OSF. PDEs could hydrolyze the cAMP and cGMP to their inactive 5′nucleotide monophosphate, 5′ AMP and 5′ GMP, respectively (Essayan, 2001), and are widely involved in the fibrosis of various tissues and organs such as heart, lung, kidney, and skin. In the kidney, PDE/cAMP/Epac/C-EBP-β signal cascade regulates renal fibrosis in renal tubular epithelial cells (Ding et al., 2018). In skin tissue, the inhibition of PDE4 induces suppression of the activity of inflammatory cells and M2 macrophages-releasing pro-fibrotic cytokines and the activation of fibroblasts and the release of collagen, therefore inhibiting dermal fibrosis (Maier et al., 2017). In alveolar epithelial cells, TGF-β1-mediated PDE4 increases enhance epithelial-mesenchymal transition (EMT), which has been regarded as a key event within the pathological mechanism of organ fibrosis (Kolosionek et al., 2009). As a cAMP-specific phosphodiesterase, PDE4 is mainly found in inflammatory cells, such as fibroblasts. The online database confirms that among the candidate factors that might interact with arecoline, PDE4A expression is significantly increased in oral tumor tissue samples. In the collected OSF tissues, PDE4A expression also increased compared to normal buccal mucosa smaples (Supplementary Figure S1). Consistently, PDE4A could be significantly induced by TGF-β1 stimulation, indicating that PDE4A might be involved in arecoline functions in TGF-β1-induced BMFs activation.

As for the molecular functions, PDE4A silence significantly reversed the promotive effects of arecoline on TGF-β1-caused BMFs activation. However, arecoline caused almost no changes in PDE4A protein levels upon TGF-β1 stimulation. At the same time, arecoline decreased, while PDE4A silence increased the cAMP concentration; the effects of arecoline on cAMP concentration could be significantly reversed by PDE4A silence upon TGF-β1 stimulation, indicating that arecoline might exert its effects on TGF-β1-induced BMFs activation via modulating PDE4A activity but not PDE4A expression. Furthermore, PDE4 specific inhibitor rolipram significantly suppressed TGF-β1-induced BMFs activation. As we have mentioned, PDE4 is considered a cAMP-specific phosphodiesterase mainly found in inflammatory cells, such as fibroblasts. Over a long period, PKA2 represented the only known effector of cAMP (Kopperud et al., 2003); nevertheless, though its activity is required, its effect on cAMP mitosis is inadequate (Dremier et al., 1997; Cass et al., 1999). Epac1 is considered another effector of cAMP, which activates its target Rap1 to enhance fibroblasts’ migratory capacity. After activation, Rap1 binds to RAPL to promote the polarization of cells via the activation of integrins (Yokoyama et al., 2008). Notably, TGF-β suppresses the expression of Epac1 in a Smad3-dependent manner (Yokoyama et al., 2008). Moreover, Epac1 overexpression suppresses TGF-β-caused collagen synthesis, suggesting pro-fibrotic response requires the downregulation in the expression of Epac (Yokoyama et al., 2008). In the present study, arecoline and PDE4A silence caused only moderate changes in cAMP-PKA signaling factors while significantly altering cAMP-Epac1 signaling factors. Moreover, Epac1-selective cAMP analog 8-Me-cAMP but not PAK-selective cAMP analog N6-cAMP remarkably suppressed α-SMA and Col1A1 protein levels upon TGF-β1 and arecoline co-treatment. These data indicate that the cAMP-Epac1 but not cAMP-PKA signaling is involved in arecoline functions in TGF-β1-induced BMFs activation.

**FIGURE 6:**
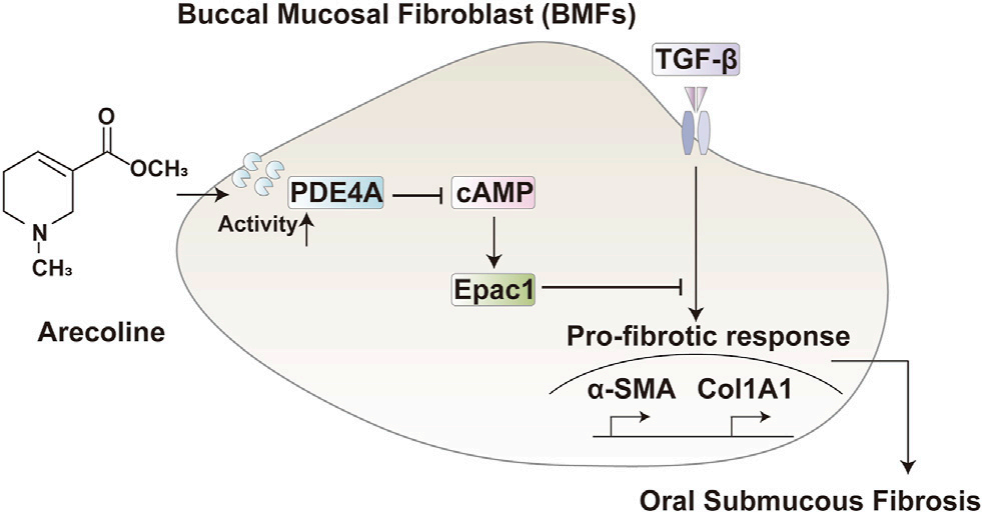
A schematic diagram. In TGF-β activated buccal mucosal fibroblast, arecoline increased the PDE4A activity, therefore inhibiting the cAMP/Epac1 pathway and promoting the oral submucous fibrosis.

In conclusion, arecoline enhances the activity of PDE4A to promote TGF-β1-induced BMFs activation via the cAMP-Epac1 signaling pathway during OSF (Figure 6). This novel mechanism indicated that blocking the cAMP-Epac1 signaling pathway is a powerful strategy for OSF therapy. However, the lack of validation in animal models and the limited clinical samples size are the main limitations of this study.

## Data Availability

Publicly available datasets were analyzed in this study. This data can be found here: GEO Date GSE107591 https://www.ncbi.nlm.nih.gov/geo/query/acc.cgi.

## Supplementary Material

Supplementary Figure S1 Expression of PDE4A in head and neck cancer based on TCGA database and clinical samples. **(A)** the expression of PDE7A, PDE6D and PDE4A in TCGA-HNSC. The number of normal and primary tumor tissues is 44 and 520, respectively. ***p* < 0.01 compared with normal tissues, Mann-Whitney U test **(B)** the histological change and expression of PDE4A in oral submucous fibrosis tissues by HE and IHC staining. *n* = 6. ***p* < 0.01 compared with normal tissues, student t-test.

Supplementary Figure S2 Arecoline affected cAMP/Epac1 pathway. BMFs were treated with 50 µg/ml arecoline alone for 48 h. **(A)** The expression of PDE4A was determined. *n* = 3. **(B)** The extracellular PDE4A activity was determined by a PDE4A assay kit s method section description. **(C)** The expression of PDE4B, PDE4C and PDE4D was determined, *n* = 3. **(D)** The expression of Epac1 was determined, *n* = 3. **(E)** The expression of PKA and p-PKA was determined, *n* = 3. ***p* < 0.01 compared with the control group, student t-test.

## References

[B1] Cass L. A. Summers S. A. Prendergast G. V. Backer J. M. Birnbaum M. J. Meinkoth J. L. (1999). Protein Kinase A-dependent and -independent Signaling Pathways Contribute to Cyclic AMP-Stimulated Proliferation. Mol. Cel Biol 19 (9), 5882–5891. 10.1128/mcb.19.9.5882 PMC84437 10454535

[B2] Chandrashekar D. S. Bashel B. Balasubramanya S. A. H. Creighton C. J. Ponce-Rodriguez I. Chakravarthi B. V. S. K. (2017). UALCAN: A Portal for Facilitating Tumor Subgroup Gene Expression and Survival Analyses. Neoplasia 19 (8), 649–658. 10.1016/j.neo.2017.05.002 28732212 PMC5516091

[B3] Chang Y. C. Tai K. W. Cheng M. H. Chou L. S. Chou M. Y. (1998). Cytotoxic and Non-genotoxic Effects of Arecoline on Human Buccal Fibroblasts *In Vitro* . J. Oral Pathol. Med. 27 (2), 68–71. 10.1111/j.1600-0714.1998.tb02096.x 9526732

[B4] Chang Y. C. Tsai C. H. Lai Y. L. Yu C. C. Chi W. Y. Li J. J. (2014). Arecoline-induced Myofibroblast Transdifferentiation from Human Buccal Mucosal Fibroblasts Is Mediated by ZEB1. J. Cel Mol Med 18 (4), 698–708. 10.1111/jcmm.12219 PMC4000120 24400868

[B5] Chang Y. C. Tsai C. H. Tai K. W. Yang S. H. Chou M. Y. Lii C. K. (2002). Elevated Vimentin Expression in Buccal Mucosal Fibroblasts by Arecoline *In Vitro* as a Possible Pathogenesis for Oral Submucous Fibrosis. Oral Oncol. 38 (5), 425–430. 10.1016/s1368-8375(01)00083-5 12110335

[B6] Csanády L. Vergani P. Gadsby D. C. (2019). Structure, Gating, and Regulation of the Cftr Anion Channel. Physiol. Rev. 99 (1), 707–738. 10.1152/physrev.00007.2018 30516439

[B7] Davis S. Meltzer P. S. (2007). GEOquery: a Bridge between the Gene Expression Omnibus (GEO) and BioConductor. Bioinformatics 23 (14), 1846–1847. 10.1093/bioinformatics/btm254 17496320

[B8] Ding H. Bai F. Cao H. Xu J. Fang L. Wu J. (2018). PDE/cAMP/Epac/C/EBP-β Signaling Cascade Regulates Mitochondria Biogenesis of Tubular Epithelial Cells in Renal Fibrosis. Antioxid. Redox Signal. 29 (7), 637–652. 10.1089/ars.2017.7041 29216750

[B9] Dremier S. Pohl V. Poteet-Smith C. Roger P. P. Corbin J. Doskeland S. O. (1997). Activation of Cyclic AMP-dependent Kinase Is Required but May Not Be Sufficient to Mimic Cyclic AMP-dependent DNA Synthesis and Thyroglobulin Expression in Dog Thyroid Cells. Mol. Cel Biol 17 (11), 6717–6726. 10.1128/mcb.17.11.6717 PMC232526 9343436

[B10] Ekanayaka R. P. Tilakaratne W. M. (2016). Oral Submucous Fibrosis: Review on Mechanisms of Malignant Transformation. Oral Surg. Oral Med. Oral Pathol. Oral Radiol. 122 (2), 192–199. 10.1016/j.oooo.2015.12.018 27289264

[B11] Essam R. M. Ahmed L. A. Abdelsalam R. M. El-Khatib A. S. (2019). Phosphodiestrase-1 and 4 Inhibitors Ameliorate Liver Fibrosis in Rats: Modulation of cAMP/CREB/TLR4 Inflammatory and Fibrogenic Pathways. Life Sci. 222, 245–254. 10.1016/j.lfs.2019.03.014 30858122

[B12] Essayan D. M. (2001). Cyclic Nucleotide Phosphodiesterases. J. Allergy Clin. Immunol. 108 (5), 671–680. 10.1067/mai.2001.119555 11692087

[B13] Gao Y. Raj J. U. (2010). Regulation of the Pulmonary Circulation in the Fetus and Newborn. Physiol. Rev. 90 (4), 1291–1335. 10.1152/physrev.00032.2009 20959617

[B14] Haider S. M. Merchant A. T. Fikree F. F. Rahbar M. H. (2000). Clinical and Functional Staging of Oral Submucous Fibrosis. Br. J. Oral Maxillofac. Surg. 38 (1), 12–15. 10.1054/bjom.1999.0062 10783440

[B15] Khan I. Kumar N. Pant I. Narra S. Kondaiah P. (2012). Activation of TGF-β Pathway by Areca Nut Constituents: a Possible Cause of Oral Submucous Fibrosis. PLoS One 7 (12), e51806. 10.1371/journal.pone.0051806 23284772 PMC3526649

[B16] Ko Y. C. Huang Y. L. Lee C. H. Chen M. J. Lin L. M. Tsai C. C. (1995). Betel Quid Chewing, Cigarette Smoking and Alcohol Consumption Related to Oral Cancer in Taiwan. J. Oral Pathol. Med. 24 (10), 450–453. 10.1111/j.1600-0714.1995.tb01132.x 8600280

[B17] Kolosionek E. Savai R. Ghofrani H. A. Weissmann N. Guenther A. Grimminger F. (2009). Expression and Activity of Phosphodiesterase Isoforms during Epithelial Mesenchymal Transition: the Role of Phosphodiesterase 4. Mol. Biol. Cel 20 (22), 4751–4765. 10.1091/mbc.e09-01-0019 PMC2777105 19759179

[B18] Kopperud R. Krakstad C. Selheim F. Døskeland S. O. (2003). cAMP Effector Mechanisms. Novel Twists for an 'old' Signaling System. FEBS Lett. 546 (1), 121–126. 10.1016/s0014-5793(03)00563-5 12829247

[B19] Liu H. Deng H. Zhao Y. Li C. Liang Y. (2018). LncRNA XIST/miR-34a axis Modulates the Cell Proliferation and Tumor Growth of Thyroid Cancer through MET-Pi3k-AKT Signaling. J. Exp. Clin. Cancer Res. 37 (1), 279. 10.1186/s13046-018-0950-9 30463570 PMC6249781

[B20] Liu Z. Guo F. Wang Y. Li C. Zhang X. Li H. (2016). BATMAN-TCM: a Bioinformatics Analysis Tool for Molecular mechANism of Traditional Chinese Medicine. Sci. Rep. 6 (1), 21146. 10.1038/srep21146 26879404 PMC4754750

[B21] Luo Z. Yi Z. J. Ou Z. L. Han T. Wan T. Tang Y. C. (2019). RELA/NEAT1/miR-302a-3p/RELA Feedback Loop Modulates Pancreatic Ductal Adenocarcinoma Cell Proliferation and Migration. J. Cel Physiol 234 (4), 3583–3597. 10.1002/jcp.27039 30362505

[B22] Maier C. Ramming A. Bergmann C. Weinkam R. Kittan N. Schett G. (2017). Inhibition of Phosphodiesterase 4 (PDE4) Reduces Dermal Fibrosis by Interfering with the Release of Interleukin-6 from M2 Macrophages. Ann. Rheum. Dis. 76 (6), 1133–1141. 10.1136/annrheumdis-2016-210189 28209630

[B23] Meghji S. Haque M. F. Harris M. (1997). Oral Submucous Fibrosis and Copper. Lancet 350 (9072), 220. 10.1016/s0140-6736(05)62388-4 9250210

[B24] Mihályi C. Iordanov I. Töröcsik B. Csanády L. (2020). Simple Binding of Protein Kinase A Prior to Phosphorylation Allows CFTR Anion Channels to Be Opened by Nucleotides. Proc. Natl. Acad. Sci. U S A. 117 (35), 21740–21746. 10.1073/pnas.2007910117 32817533 PMC7474675

[B25] More C. B. Jatti Patil D. Rao N. R. (2020). Medicinal Management of Oral Submucous Fibrosis in the Past Decade- A Systematic Review. J. Oral Biol. Craniofac. Res. 10 (4), 552–568. 10.1016/j.jobcr.2020.08.004 32939334 PMC7479289

[B26] More C. B. Rao N. R. (2019). Proposed Clinical Definition for Oral Submucous Fibrosis. J. Oral Biol. Craniofac. Res. 9 (4), 311–314. 10.1016/j.jobcr.2019.06.016 31334003 PMC6614531

[B27] Moutasim K. A. Jenei V. Sapienza K. Marsh D. Weinreb P. H. Violette S. M. (2011). Betel-derived Alkaloid Up-Regulates Keratinocyte Alphavbeta6 Integrin Expression and Promotes Oral Submucous Fibrosis. J. Pathol. 223 (3), 366–377. 10.1002/path.2786 21171082

[B28] Ni W. F. Tsai C. H. Yang S. F. Chang Y. C. (2007). Elevated Expression of NF-kappaB in Oral Submucous Fibrosis-Eevidence for NF-kappaB Induction by Safrole in Human Buccal Mucosal Fibroblasts. Oral Oncol. 43 (6), 557–562. 10.1016/j.oraloncology.2006.06.007 16996785

[B29] Nishimura G. Manabe I. Tsushima K. Fujiu K. Oishi Y. Imai Y. (2006). DeltaEF1 Mediates TGF-Beta Signaling in Vascular Smooth Muscle Cell Differentiation. Dev. Cel 11 (1), 93–104. 10.1016/j.devcel.2006.05.011 16824956

[B30] Qin D. Zhang H. Zhang H. Sun T. Zhao H. Lee W. H. (2019). Anti-osteoporosis Effects of Osteoking via Reducing Reactive Oxygen Species. J. Ethnopharmacol 244, 112045. 10.1016/j.jep.2019.112045 31260757

[B31] Rao N. R. Villa A. More C. B. Jayasinghe R. D. Kerr A. R. Johnson N. W. (2020). Oral Submucous Fibrosis: a Contemporary Narrative Review with a Proposed Inter-professional Approach for an Early Diagnosis and Clinical Management. J. Otolaryngol. Head Neck Surg. 49 (1), 3. 10.1186/s40463-020-0399-7 31915073 PMC6951010

[B32] Ritchie M. E. Phipson B. Wu D. Hu Y. Law C. W. Shi W. (2015). Limma powers Differential Expression Analyses for RNA-Sequencing and Microarray Studies. Nucleic Acids Res. 43 (7), e47. 10.1093/nar/gkv007 25605792 PMC4402510

[B33] Shieh D. H. Chiang L. C. Shieh T. Y. (2003). Augmented mRNA Expression of Tissue Inhibitor of Metalloproteinase-1 in Buccal Mucosal Fibroblasts by Arecoline and Safrole as a Possible Pathogenesis for Oral Submucous Fibrosis. Oral Oncol. 39 (7), 728–735. 10.1016/s1368-8375(03)00101-5 12907213

[B34] Syed A. U. Koide M. Brayden J. E. Wellman G. C. (2019). Tonic Regulation of Middle Meningeal Artery Diameter by ATP-Sensitive Potassium Channels. J. Cereb. Blood Flow Metab. 39 (4), 670–679. 10.1177/0271678X17749392 29260608 PMC6446425

[B35] Szklarczyk D. Franceschini A. Wyder S. Forslund K. Heller D. Huerta-Cepas J. (2015). STRING V10: Protein-Protein Interaction Networks, Integrated over the Tree of Life. Nucleic Acids Res. 43 (Database issue), D447–D452. 10.1093/nar/gku1003 25352553 PMC4383874

[B36] Tilakaratne W. M. Klinikowski M. F. Saku T. Peters T. J. Warnakulasuriya S. (2006). Oral Submucous Fibrosis: Review on Aetiology and Pathogenesis. Oral Oncol. 42 (6), 561–568. 10.1016/j.oraloncology.2005.08.005 16311067

[B37] Tsai C. H. Yang S. F. Chen Y. J. Chou M. Y. Chang Y. C. (2005). The Upregulation of Insulin-like Growth Factor-1 in Oral Submucous Fibrosis. Oral Oncol. 41 (9), 940–946. 10.1016/j.oraloncology.2005.05.006 16054426

[B38] Waldkirch E. S. Ückert S. Sigl K. Satzger I. Geismar U. Langnäse K. (2010). Expression of Cyclic AMP-dependent Protein Kinase Isoforms in Human Cavernous Arteries: Functional Significance and Relation to Phosphodiesterase Type 4. J. Sex. Med. 7 (6), 2104–2111. 10.1111/j.1743-6109.2010.01808.x 20487244

[B39] Wang J. Jiang C. Li N. Wang F. Xu Y. Shen Z. (2020). The circEPSTI1/mir-942-5p/LTBP2 axis Regulates the Progression of OSCC in the Background of OSF via EMT and the PI3K/Akt/mTOR Pathway. Cell Death Dis 11 (8), 682. 10.1038/s41419-020-02851-w 32826876 PMC7443145

[B40] Wójcik-Pszczoła K. Chłoń-Rzepa G. Jankowska A. Ślusarczyk M. Ferdek P. E. Kusiak A. A. (2020). A Novel, Pan-PDE Inhibitor Exerts Anti-fibrotic Effects in Human Lung Fibroblasts via Inhibition of TGF-β Signaling and Activation of cAMP/PKA Signaling. Int. J. Mol. Sci. 21 (11), 4008. 10.3390/ijms21114008 PMC7312375 32503342

[B41] Wollina U. Verma S. B. Ali F. M. Patil K. (2015). Oral Submucous Fibrosis: an Update. Clin. Cosmet. Investig. Dermatol. 8, 193–204. 10.2147/CCID.S80576 PMC4401336 25914554

[B42] Yang S. F. Hsieh Y. S. Tsai C. H. Chou M. Y. Chang Y. C. (2003). The Upregulation of Type I Plasminogen Activator Inhibitor in Oral Submucous Fibrosis. Oral Oncol. 39 (4), 367–372. 10.1016/s1368-8375(02)00123-9 12676256

[B43] Yokoyama U. Patel H. H. Lai N. C. Aroonsakool N. Roth D. M. Insel P. A. (2008). The Cyclic AMP Effector Epac Integrates Pro- and Anti-fibrotic Signals. Proc. Natl. Acad. Sci. U S A. 105 (17), 6386–6391. 10.1073/pnas.0801490105 18434542 PMC2359804

[B44] Yu C. C. Tsai C. H. Hsu H. I. Chang Y. C. (2013). Elevation of S100A4 Expression in Buccal Mucosal Fibroblasts by Arecoline: Involvement in the Pathogenesis of Oral Submucous Fibrosis. PLoS One 8 (1), e55122. 10.1371/journal.pone.0055122 23383075 PMC3561403

[B45] Yu C. C. Tsai L. L. Wang M. L. Yu C. H. Lo W. L. Chang Y. C. (2013). miR145 Targets the SOX9/ADAM17 axis to Inhibit Tumor-Initiating Cells and IL-6-mediated Paracrine Effects in Head and Neck Cancer. Cancer Res. 73 (11), 3425–3440. 10.1158/0008-5472.CAN-12-3840 23548270

[B46] Yu C. C. Yu C. H. Chang Y. C. (2016). Aberrant SSEA-4 Upregulation Mediates Myofibroblast Activity to Promote Pre-cancerous Oral Submucous Fibrosis. Sci. Rep. 6, 37004. 10.1038/srep37004 27845370 PMC5109465

[B47] Zhang H. T. (2009). Cyclic AMP-specific Phosphodiesterase-4 as a Target for the Development of Antidepressant Drugs. Curr. Pharm. Des. 15 (14), 1688–1698. 10.2174/138161209788168092 19442182

[B48] Znaor A. Brennan P. Gajalakshmi V. Mathew A. Shanta V. Varghese C. (2003). Independent and Combined Effects of Tobacco Smoking, Chewing and Alcohol Drinking on the Risk of Oral, Pharyngeal and Esophageal Cancers in Indian Men. Int. J. Cancer 105 (5), 681–686. 10.1002/ijc.11114 12740918

