# Supplementary material for: Arecoline Enhances Phosphodiesterase 4A Activity to Promote Transforming Growth Factor-β-Induced Buccal Mucosal Fibroblast Activation via cAMP-Epac1 Signaling Pathway
Source: Front Pharmacol. 2021 Nov 8;12:722040. doi: 10.3389/fphar.2021.722040 (PMC8606562; doi:10.3389/fphar.2021.722040)
Supplement: Supplementary file 1 [file Table1.DOCX]

**Table S1 Phosphodiesterase family members interacting with arecoline**

| **NCBI gene ID** | **Gene Name** | **Score** |
| --- | --- | --- |
| 5136 | PDE1A | 8.253 |
| 5153 | PDE1B | 8.253 |
| 27115 | PDE7B | 2.328 |
| 8654 | PDE5A | 2.328 |
| 5152 | PDE9A | 2.328 |
| **5141** | **PDE4A** | **2.328** |
| 201626 | PDE12 | 2.328 |
| 5148 | PDE6G | 2.328 |
| 5139 | PDE3A | 2.328 |
| 5145 | PDE6A | 2.328 |
| 5140 | PDE3B | 2.328 |
| 5144 | PDE4D | 2.328 |
| 9659 | PDE4DIP | 2.328 |
| 5150 | PDE7A | 2.328 |
| 5143 | PDE4C | 2.328 |
| 10846 | PDE10A | 2.328 |
| 5137 | PDE1C | 2.328 |
| 5147 | PDE6D | 2.328 |
| 5158 | PDE6B | 2.328 |
| 5142 | PDE4B | 2.328 |
| 5138 | PDE2A | 2.328 |
| 5149 | PDE6H | 2.328 |
