# Supplementary figures and images for: Arecoline Enhances Phosphodiesterase 4A Activity to Promote Transforming Growth Factor-β-Induced Buccal Mucosal Fibroblast Activation via cAMP-Epac1 Signaling Pathway
Source: Front Pharmacol. 2021 Nov 8;12:722040. doi: 10.3389/fphar.2021.722040 (PMC8606562; doi:10.3389/fphar.2021.722040)

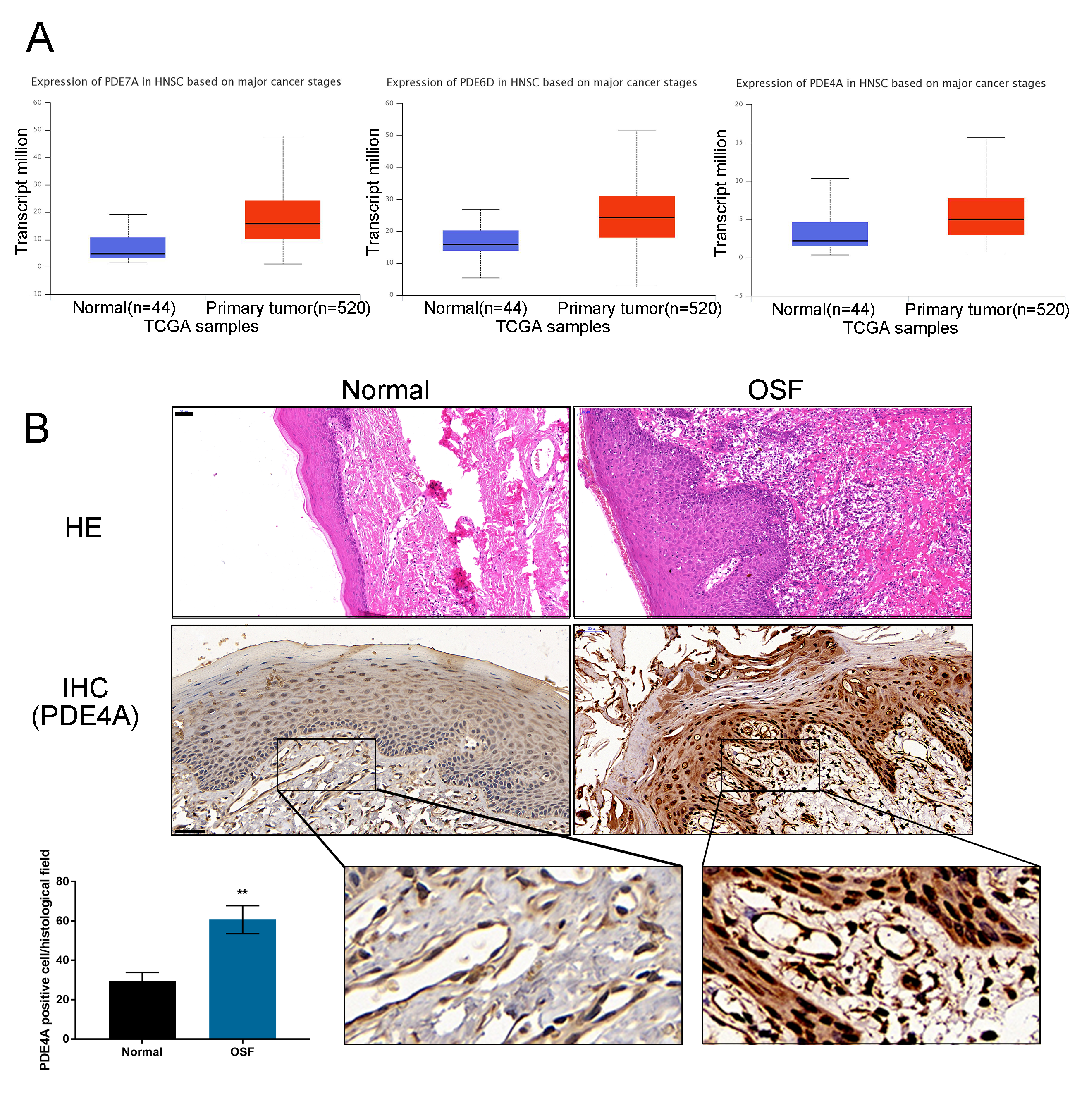

Supplement: Supplementary file 2 [file Image2.TIF]

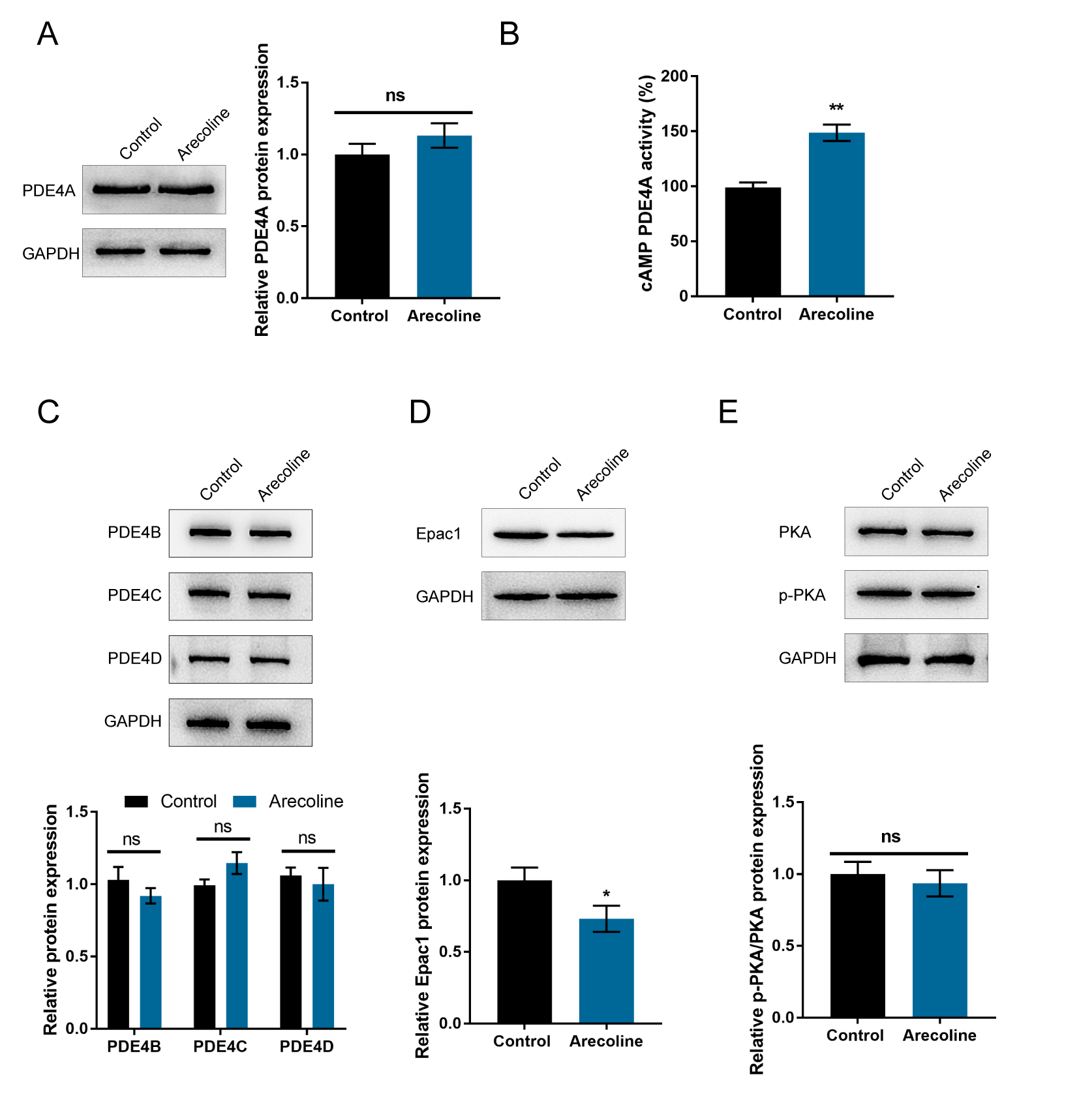

Supplement: Supplementary file 3 [file Image1.tif]
