# Supplementary material for: Arecoline Enhances Phosphodiesterase 4A Activity to Promote Transforming Growth Factor-β-Induced Buccal Mucosal Fibroblast Activation via cAMP-Epac1 Signaling Pathway
Source: Front Pharmacol. 2021 Nov 8;12:722040. doi: 10.3389/fphar.2021.722040 (PMC8606562; doi:10.3389/fphar.2021.722040)
Supplement: Supplementary file 4 [file Table2.DOCX]

Table S2 Differential expression of PDE family in clinical oral cancer samples (GSE107591)

| **adj.P.Val** | **P.Value** | **t** | **B** | **logFC** | **Gene.symbol** |
| --- | --- | --- | --- | --- | --- |
| 4.42E-04 | 5.45E-06 | -5.1026781 | 3.891227 | -0.6642692 | PDE2A |
| **7.88E-03** | **3.16E-04** | **3.8761658** | **0.07067** | **0.6365299** | **PDE4A** |
| 9.93E-03 | 4.38E-04 | -3.7714297 | -0.233325 | -0.3449429 | PDE12 |
| 9.99E-03 | 4.42E-04 | -3.7684017 | -0.242048 | -0.6407487 | PDE1A |
| 1.30E-02 | 6.40E-04 | -3.648128 | -0.585448 | -0.6250051 | PDE6A |
| 2.29E-02 | 1.43E-03 | 3.380778 | -1.325819 | 0.8108337 | PDE7A |
| 1.81E-01 | 3.15E-02 | -2.2141041 | -4.095891 | -0.4361743 | PDE5A |
| 1.95E-01 | 3.56E-02 | -2.1607309 | -4.201288 | -0.3410808 | PDE4D |
| 2.03E-01 | 3.78E-02 | 2.1346544 | -4.252015 | 0.3777111 | PDE3A |
| 2.14E-01 | 4.14E-02 | -2.0943574 | -4.329403 | -0.2448524 | PDE9A |

t: T value of Bayesian. t.test; B: log transformation of the standard deviation value of Bayesian. t.test.
